# Supplementary material for: Exploring barriers to parent-adolescent sexual-risk communication among adolescents in Port Harcourt Nigeria: Adolescents’ and parents’ perspective
Source: PLOS Glob Public Health. 2025 Jan 21;5(1):e0003148. doi: 10.1371/journal.pgph.0003148 (PMC11750103; doi:10.1371/journal.pgph.0003148)
Supplement: S1 Text — (DOCX) [file pgph.0003148.s001.docx]

**S1 Text**

**Data A. FGD Transcribed (Males)**

Adolescent males: ages 15-19

Respondents were assigned numbers for easy identification such as M1, M2, M3 ………..M8

Ages of Adolescents

M1: 18 M5: 16

M2: 17 M6: 17

M3: 16 M7: 18

M4: 18 M8: 15

Have you ever received sex education from your parents? If No, who/where did u receive it?

M1- No, from school

M2- No, from Church

M3- Yes I have

M4- No, from Church

M5- Yes I have

M6- Yes I have received from them

M7- Yes I have from them

M8- No, from Church

Do your parents discuss sex education with you often?

M1- Never

M2- Never

M3- Sometimes

M4- Never

M5- Rarely

M6- Often

M7- Rarely

M8- Never

What do you think hinders your parents from effectively discussing with you on sex education?

**Fear/discomfort with discussion**:

M5- My parents rarely discuss sexual issues with me because they are always so uncomfortable with the discussion which also makes me uncomfortable too. I think we all noticed this and stopped its discussion completely. They don’t discuss sexual matters with me anymore.

M4- I would love to discuss sexual issues with my parents but I am scared of what they will think or feel about me.

**Belief that it will initiate sex**: 2 Respondents

M1- Whatever information I got first about sex was from the school. My parents have never discussed sexual issues with me because they probably feel that I would want to venture into sex.

M2- The first I asked my dad about sex, he asked me if I had already started having sex or want to have sex. He was so angry that day that I never asked him again.

**Parents are ignorant or unaware**: Nil

**Parents are too busy**: 3 Respondents

M3- My dad is hardly ever home and even when he is, he says he’s too tired to have any conversations with me. All of my sexual knowledge is either from school, my friends or from the internet.

M8- My Dad is a big business man. He’s always busy. Mum refuses to engage in conversations on sex education. She says I should go meet my Dad. I learn what I can from the internet and friends too.

M5- My mother and father are both traders that work Mondays to Saturdays. They come home late most nights.

**Religious/cultural factor**: 1 Respondent

M6- My parents do not discuss sexual issues with me or my siblings because it is against our religion. Our Church forbids such discussions in the home. Whatever I know about sexual issues, I got from my friends in school.

**Parents are too judgmental**: 2 Respondents

M3- My dad assumed that I had already started having sex when I first asked him about condoms. He almost beat me up because of that one simple question. I was only 14 at the time and as a result I never discuss sexual issues with my dad and he doesn’t discuss with me either. I learn from peers and the internet.

M7- My first knowledge about SRH health was from school. My parents are the kind of people that read meaning into everything; even mere questions. I asking questions about these issues will mean that I have already started having sex or I want to have sex. There was this time I asked my dad if condoms were 100% effective in preventing pregnancy....My dad called my mom immediately and they started asking me all sorts of really embarrassing questions. Since then, I never asked them such questions again.

**Poor/inaccurate knowledge on sex education**: 1 respondent

M5- I don’t discuss sexual issues with my parents especially my mom because they are both ‘old school’. Whatever information they have will be archaic. It’s either I discuss with peers/friends or use the internet.

**Lack of trust**: All Respondents

M1- My Mom will report me to my Dad if I ask her about anything related to sex. She will feel that I have already started having sex.

(M8, M5, M3, M2, M6, M4, M7) - My Parents do not trust will definitely assume that I have started having sex if I ask them about sexual matters.

Do you know what risky sexual behaviors are? All male respondents said, ‘No’.

A definition of risky sexual behavior was given to the Respondent. Risky sexual behaviour according to the Center for Disease Control and prevention (CDC) is defined as a behavior that increases one’s risk of contracting Sexually Transmitted Infections (STIs) and experiencing unintended pregnancy.

Do you think risky sexual behaviors can be prevented if adolescents received sex education from schools or parents?

M1- Yes, if adequate information on RSB and sexual education will lead to adolescents making the right sexual decisions like practicing safe sex.

M2- Yes. Adolescents may not want to indulge in RSB because they are risky and may harm them somehow. No, probably due to peer pressure/curiosity. Friends influence us easily and because our friends are doing it, we may want to join them so as to ‘feel among’.

M3- Risky sexual behavior can be prevented if we receive adequate sexual education. Having adequate information will lead us to making the right decisions.

M4- Yes, it could be prevented because the right knowledge will lead to taking the right decisions. Probably because teenagers are generally curious and may want to experiment sexually.

M5- Risky sexual behavior can be prevented or reduced if adolescents have adequate sexual information which will in turn prevent adolescents from indulging in RSB and make the right decisions with regards to sexual health.

M6- Yes because no one wants to be a victim of wrong sexual decisions especially sexual decisions that affect one’s health. Having adequate information will lead to limited RSB.

M7- Yes. When we have the right sexual education and information, we are less likely to venture into RSB.

M8- It won’t have any effect because adolescents do not listen and they also like experimenting in things that may or may not be harmful.

**Data B. FGD Transcribed (Females)**

Adolescent females (15-19 years): 8 Students

Respondents were assigned numbers for easy identification such as F1, F2, F3 ………..F8

Ages of adolescents

F1: 15 F5: 16

F2: 15 F6: 17

F3: 16 F7: 18

F4: 16 F8: 17

Have you ever received sex education form your parents? If No, who/where did u receive it?

F1- Yes

F2- Yes

F3- Yes

F4- No, from school

F5- No, from school

F6- Yes

F7- Yes

F8- No, from friends

Do your parents discuss sex education with you often?

F1- Sometimes

F2- Often

F3- Often

F4- Never

F5- Never

F6- Often

F7- Often

F8- Never

What do you think hinders your parents from effectively discussing with you on sex education?

**Fear/discomfort with discussion:** 4 Respondents

F1- Discussing sex with my parents is a ‘no-go’ area.

F4- I’m not very close to my Mom so, discussion about this would be uncomfortable for both of us.

F3- I don’t like having topics on sexual issues.

F8- Whenever I ask my Mom about sexual issues, she gets so uncomfortable with the discussion that after a while I stopped discussing with her but I discuss with my friends in school.

**Belief that it will initiate sex**: 1 Respondent

F5- My mother will think that I have a boyfriend if I ask her about sex. She will most likely give me a slap or a beating.

**Parents are ignorant or unaware**: Nil

**Parents are too busy**: 2 Respondents

F2- My mom is always travelling for business so, discussing sex with her is almost impossible. I can’t discuss with my dad because he is of the opposite sex. Most of the knowledge I have on sex is what I was taught in school by my teachers, some form my friends too.

F6- My mother is always working. We barely discuss anything. Whenever I want to engage in a conversation, she says, ‘she needs to rest’.

**Religious/cultural factor**: 1 Respondent

F2- Whenever I ask my mom about issues relating to sex, she always tells me it is against our culture to discuss such issues with children.

**Parents are too judgmental**: All Respondents

F8- I don’t discuss sexual matters with my mom because she will just assume that I want to start having sex that I why I am asking so, I don’t bother meeting her to start up such discussions.

F4- My parents are just too religious. Such discussion is a forbidden at home. If I even ask them by mistake, they will skin me alive because they will assume that I want to start experimenting. I’m not even allowed to have male friends around me.

F1, F2, F3, F5, F6, & F7- My Mom is so judgmental. I can never discuss such with her.

**Poor/inaccurate knowledge on sex education**: All Respondents

F2- I don’t think my parents are well equipped to give me adequate knowledge on SRH issues. They do not just have enough knowledge. At least I receive up-to-date information in school from my teachers, peers and sometimes from the internet.

F1, F3, F5, F6, & F8 - I don’t think my parents have the current knowledge on sex education.

**Lack of trust**: All Respondents

F4- My Mom just doesn’t trust me to have these kinds of discussions with me. She feels having these discussions is a way of encouraging me to venture into sex which isn’t true at all.

F1- My parents do not trust my friends because they feel they will influence me negatively and as a result they do not trust me either. I have been warned against having a boyfriend or having sex. If I dare broach the subject of sex to them, then I’m looking for trouble.

F6- I discuss more of SRH issues like physical development, menstruation with my mom because we are of the same gender. I dare not mention sex or any related topic to her because she will beat me. She will think that I have a boyfriend and want to experiment with him.

The respondents were asked what risky sexual behaviours are?

Risky sexual behaviors were defined for the respondent and they were asked if they have ever indulged. Risky sexual behaviour according to the Center for Disease Control and prevention (CDC) is defined as a behavior that increases one’s risk of contracting Sexually Transmitted Infections (STIs) and experiencing unintended pregnancy.

Respondent weren’t honest with answers so I switched to the next question.

Do you think risky sexual behaviors can be prevented if adolescents received sex education from schools or parents?

F1- Yes it will be prevented/reduced if we receive adequate sexual education from our parents on various topics that will help us make the right decisions especially sexual decisions.

F2- It won’t really be reduced because adolescents are generally curious and will want to indulge or experiment in sexual activities.

F3- Yes and No. RSB can be prevented because most adolescents listen to their parents and follow the advice of their parents more than any other person.

F3- No, because the curiosity of adolescents will make them want to indulge. Other factors include family background or upbringing, peer pressure and other family members are doing it.

F4- Limited effect because most teens do not listen to their parents, they are too curious and will want to experiment. Peer pressure from friends that are indulging in sexual activities will also make adolescents want to indulge. TV programmes/movies that depict sex can make them also want to experiment.

F5- No. Adolescents are too curious and will want to indulge in sexual activities whether risky or not.

F6- No. Peer pressure and ignorance of RSB will move adolescents to exploit sexually especially when adequate sexual education wasn’t received.

F7- Yes because adolescents listen to their parents and will wait for the right time to have sex. F8- Yes. If parents talk calmly and freely, adolescents won’t indulge and ‘No’ because parents being strict and controlling will make them want to indulge as a form of rebellion.
