# Supplementary material for: Exploring barriers to parent-adolescent sexual-risk communication among adolescents in Port Harcourt Nigeria: Adolescents’ and parents’ perspective
Source: PLOS Glob Public Health. 2025 Jan 21;5(1):e0003148. doi: 10.1371/journal.pgph.0003148 (PMC11750103; doi:10.1371/journal.pgph.0003148)
Supplement: S2 Text — Transcribed data for adolescent participant (15–19 years). Responses to FGD questions raised. Transcription of IDI question responses for parents of adolescents. (DOCX) [file pgph.0003148.s002.docx]

**S2 Text. IDI for Parents of Adolescents**

**Data A. IDI for P1**

Age: Male (51-55years)

Occupation: Trader

Marital Status: Married

Location: School Premises

Interviewer: What is your knowledge on Sexual Reproductive Health or Sex education?

N.B. An explanation was given on sex education

Respondent: I don’t know what you mean

Interviewer: Do you think parents should discuss sex education with their children?

Respondent: “No, discussion sex with my children will give them ideas to experiment. I can’t discuss anything that relates to sex with my children.”

Interviewer: Have you ever discussed SRH issues with your adolescents?

Why haven’t you?

Respondent: I have never discussed sex education with my children because I feel they shouldn’t know about these kinds of things. What they don’t know will not ‘spoil’ them.

Interviewer: What topics on sexual reproductive health do you discuss with your children? What topics do you consider most important?

Respondent: None (respondent has never discussed this with children)

Interviewer: Name the challenges you face in talking to your adolescent children about Sex and Reproductive Health?

Respondent: For me it’s against my tradition to discuss these things with my children especially my son who is too young. He’s only 15. I feel he should know stuff like these when he’s officially an adult”.

Interviewer: What do you think will make it easier for you as a parent to discuss SRH issues with your adolescents?

Respondent: I don’t know

Interviewer: What SRH information would you like your adolescent to receive at school?

Respondent: I don’t know

Interviewer: How do you think parents can be encouraged to participate in programmes on SRH in the school?

Respondent: I don’t know

Interviewer: Do you wish to make any comments or ask questions regarding the discussed topic?

Respondent: No

**Data B. IDI for P2**

Age: Male (41-45years)

Occupation: Teacher

Marital Status: Married

Location: School Premises

Interviewer: What is your knowledge on Sexual Reproductive Health or Sex education?

Respondent: I’m a teacher and sex education is part of the school curricula. I teach it to my students because I’m required to as a Biology teacher so, I understand what sex education is.

Interviewer: Do you think parents should discuss sex education with their children?

Respondent: Even though the school teaches them about puberty, parents should do same in addition to sex education for their children.

Interviewer: Have you ever discussed SRH issues with your adolescents?

Respondent: Of course I have

Interviewer: What topics on sexual reproductive health do you discuss with your children? What topics do you consider most important?

Respondent: Puberty and sex education. These are important topics.

Interviewer: Name the challenges you face in talking to your adolescent children about Sex and Reproductive Health?

Respondent: I didn’t face any challenges

Interviewer: What do you think will make it easier for you as a parent to discuss SRH issues with your adolescents?

Respondent: I don’t have difficulty discussing sex education with my children. I teach it in school so, that makes it easier for me.

Interviewer: What SRH information would you like your adolescent to receive at school?

Respondent: Puberty, menstruation for the females, STIs and STDs, safe sex.

Interviewer: How do you think parents can be encouraged to participate in programmes on SRH in the school?

Respondent: Discussing with them during PTA meetings.

Interviewer: Do you wish to make any comments or ask questions regarding the discussed topic?

Respondent: None, thank you

**Data C. IDI for P3**

Age: Male (41-45 years)

Occupation: Teacher

Marital Status: Married

Location: School Premises

Interviewer: What is your knowledge on Sexual Reproductive Health or Sex education?

Respondent: I know about sex education. I would say adequate knowledge

Interviewer: Do you think parents should discuss sex education with their children?

Respondent: Yes, I do

Interviewer: Have you ever discussed SRH issues with your adolescents?

Respondent: I have. My wife does too.

Interviewer: What topics on sexual reproductive health do you discuss with your children? What topics do you consider most important?

Respondent: Puberty, relationships with the opposite sex, STDs. I want my children to be knowledgeable about these things

Interviewer: Name the challenges you face in talking to your adolescent children about Sex and Reproductive Health?

Respondent: I don’t think I face any challenges.

Interviewer: What do you think will make it easier for you as a parent to discuss SRH issues with your adolescents?

Respondent: If my children initiated the discussion probably.

Interviewer: What SRH information would you like your adolescent to receive at school?

Respondent: Everything: Puberty, menstruation, sexual relationships, STDs and STIs.

Interviewer: How do you think parents can be encouraged to participate in programmes on SRH in the school?

Respondent: I am a teacher and in the course of my work, I discovered that most parents do not discuss sexual matters with their children unless something bad happens. I think schools should include parents in sex education especially during PTA meetings. Parents can then be encouraged to discuss sexual matters with their children.

Interviewer: Do you wish to make any comments or ask questions regarding the discussed topic?

Respondent: No, thank you.

**Data D. IDI for P4**

Age: Female (36 -40 years)

Occupation: Trader

Marital Status: Married

Location: School Premises

Interviewer: What is your knowledge on Sexual Reproductive Health or Sex education?

Respondent: A little bit.

Interviewer: Do you think parents should discuss sex education with their children?

Respondent: Yes, I don’t discuss with my children though because I am not comfortable having such discussion with them.

Interviewer: Have you ever discussed SRH issues with your adolescents?

Respondent: No, I don’t discuss with my children though because I am not comfortable having such discussions with them.

Interviewer: What topics on sexual reproductive health do you discuss with your children? What topics do you consider most important?

Respondent: I have never discussed sex with my children because they shouldn’t know about sex at their age. They are too young for that.

Interviewer: Name the challenges you face in talking to your adolescent children about Sex and Reproductive Health?

Respondent: My tradition forbids discussion with children on sexual issues until they are old enough for marriage. My children are too young presently to know about these things.

Interviewer: What do you think will make it easier for you as a parent to discuss SRH issues with your adolescents?

Respondent: I’m not sure.

Interviewer: What SRH information would you like your adolescent to receive at school?

Respondent: The basics: Puberty

Interviewer: How do you think parents can be encouraged to participate in programmes on SRH in the school?

Respondent: “Even though I don’t discuss sexual matters with my children, I feel that if schools invite parents over and discuss this with them, they will be more open to also discuss with their children.

Interviewer: Do you wish to make any comments or ask questions regarding the discussed topic?

Respondent: None

**Data E. IDI for P5**

Age: Female (41-45 years)

Occupation: Teacher

Marital Status: Married

Location: School Premises

Interviewer: What is your knowledge on Sexual Reproductive Health or Sex education?

Respondent: I know about it.

Interviewer: Do you think parents should discuss sex education with their children?

Respondent: Discussion with children is very necessary because parents underplay the influence they have on their children’s decisions.

Interviewer: Have you ever discussed SRH issues with your adolescents?

Respondent: No. Discussing issues like condom use, sex, STIs/STDs will make my sons feel that they can venture into sex even before they come of age. This is why I don’t discuss these with them. At least when they turn 18.

Interviewer: What topics on sexual reproductive health do you discuss with your children? What topics do you consider most important?

Respondent: The most important topics for me are abstinence, STIs/STDs, consequences of early pregnancy, relationship with the opposite sex. This is what I discuss with my girls as often as I can.

Interviewer: Name the challenges you face in talking to your adolescent children about Sex and Reproductive Health?

Respondent: “I have 2 sons who I can’t communicate on sex, condom use or things like ‘wet dreams’ because it is shameful for me. I leave this discussion for my husband to do.”

Interviewer: What do you think will make it easier for you as a parent to discuss SRH issues with your adolescents?

Respondent: It will probably be easier when they turn 18 and are more mature.

Interviewer: What SRH information would you like your adolescent to receive at school?

Respondent: sexual intercourse, condom use, menstruation, abstinence, relationships. I try to learn as much as u I can so that I can pass off this knowledge to my children.

Interviewer: How do you think parents can be encouraged to participate in programmes on SRH in the school?

Respondent: Schools should incorporate adolescent sex education during PTA sessions.

Interviewer: Do you wish to make any comments or ask questions regarding the discussed topic?

Respondent: None.

**Data F. IDI for P6**

Age: Female (36-40 years)

Occupation: Civil Servant (Cleaner)

Marital Status: Widowed

Location: School Premises

Interviewer: What is your knowledge on Sexual Reproductive Health or Sex education?

Respondent: A little

Interviewer: Do you think parents should discuss sex education with their children?

Respondent: I learnt a little from my mom when I was old enough for marriage. I don’t know why she didn’t discuss with me earlier. Maybe she wasn’t comfortable with such discussion”

Interviewer: Have you ever discussed SRH issues with your adolescents?

Respondent: I don’t have that kind of time. My husband is late so, the whole responsibility of catering for the family falls on me. It’s not easy to be the only parent, and I’m so busy. My child is also a male so; discussing SRH issues with him is not something I’m comfortable with.

Interviewer: What topics on sexual reproductive health do you discuss with your children? What topics do you consider most important?

Respondent: I have never discussed sex education with my son. I don’t think it’s important that he knows about sex. He may want to experiment if I start discussing with him.

Interviewer: Name the challenges you face in talking to your adolescent children about Sex and Reproductive Health?

Respondent: I don’t discuss with my adolescent son.

Interviewer: What do you think will make it easier for you as a parent to discuss SRH issues with your adolescents?

Respondent: I don’t know.

Interviewer: What SRH information would you like your adolescent to receive at school?

Respondent: Just the usual topics on puberty.

Interviewer: How do you think parents can be encouraged to participate in programmes on SRH in the school?

Respondent: My son is too young to know about sex but if the school invites parents to teach us SRH matters in a meeting, I may attend.

Interviewer: Do you wish to make any comments or ask questions regarding the discussed topic?

Respondent: None.

**Data G. IDI for P7**

Age: Female (36-40 years)

Occupation: Petty Trader

Marital Status: Married

Interviewer: What is your knowledge on Sexual Reproductive Health or Sex education?

Respondent: Moderate knowledge.

Interviewer: Do you think parents should discuss sex education with their children?

Respondent: Discussing things like pregnancy and prevention, relationships etc. will probably make my daughter feel that it’s okay to have sex in her relationships with the opposite sex when she’s older. Children of this age just want to experiment and besides, she is also too young to understand.

Interviewer: Have you ever discussed SRH issues with your adolescents?

Respondent: No, I haven’t. My daughter is too young for this sort of discussion and I don’t really feel comfortable discussing these kinds of things with her. How can I start educating my 11 year old daughter about pregnancy, sex or even STIs? I can’t discuss these with her at such a young age.”

Interviewer: What topics on sexual reproductive health do you discuss with your children? What topics do you consider most important?

Respondent: I have never discussed sexual issues with my children because the church frowns at it. I only discuss things like menstruation with my daughter because she needs to know about it and physical development for all my children because they all need to know.

Interviewer: Name the challenges you face in talking to your adolescent children about Sex and Reproductive Health?

Respondent: I have never discussed things like puberty with its associated changes in males and females with my children because I feel so shamed discussing these issues with them. Sex education for children is an abomination in my culture too. My mom never discussed with me so, I never do with my children,

Interviewer: What do you think will make it easier for you as a parent to discuss SRH issues with your adolescents?

Respondent: Maybe when my daughter is mature then, it will be easier.

Interviewer: What SRH information would you like your adolescent to receive at school?

Respondent: Menstruation and puberty.

Interviewer: How do you think parents can be encouraged to participate in programmes on SRH in the school?

Respondent: I don’t know.

Interviewer: Do you wish to make any comments or ask questions regarding the discussed topic?

Respondent: No.

**Data H. IDI for P8**

Age: Male (46- 50 years)

Occupation: Civil Servant

Marital Status: Married

Location: School Premises

Interviewer: What is your knowledge on Sexual Reproductive Health or Sex education?

Respondent: Adequate knowledge

Interviewer: Do you think parents should discuss sex education with their children?

Respondent: I think parents should discuss with their children. My neighbor’s daughter got pregnant at 16 and that brought shame to the family. She had to quit going to school at a point as a result of this. Maybe, if her parents had discussed these things with her, she won’t have made such a mistake. As a result of this, I had to start discussing sex with my children.

Interviewer: Have you ever discussed SRH issues with your adolescents?

Respondent: Not often but my wife and I have.

Interviewer: What topics on sexual reproductive health do you discuss with your children? What topics do you consider most important?

Respondent: I tell my children especially my daughter about bad men that may want to touch them indecently. I told them to report to me if they come across such persons. I tell my son to abstain from sex until he is old enough.

Interviewer: Name the challenges you face in talking to your adolescent children about Sex and Reproductive Health?

Respondent: I feel shy sometimes but I still do it because it’s important.

Interviewer: What do you think will make it easier for you as a parent to discuss SRH issues with your adolescents?

Respondent: I’m not sure.

Interviewer: What SRH information would you like your adolescent to receive at school?

Respondent: I know about puberty, physical development, menstruation because I was taught when I was taught it in secondary school. These topics are fine.

Interviewer: How do you think parents can be encouraged to participate in programmes on SRH in the school?

Respondent: The school should invite parents, teach and discuss sex education with them so that parents know what to teach their children at home.”

Interviewer: Do you wish to make any comments or ask questions regarding the discussed topic?

Respondent: I wish sex education was part of every school’s curricula in Nigeria. Our children would make wise sexual decisions.

**Data I. IDI for P9**

Age: Male (46-50 years)

Occupation: Civil Servant

Marital Status: Married

Interviewer: What is your knowledge on Sexual Reproductive Health or Sex education?

Respondent: A read a lot and I’ve come across it a couple of times.

Interviewer: Do you think parents should discuss sex education with their children?

Respondent: Of course. Children should be armed with the knowledge to make wise decisions regarding their sexual life”

Interviewer: Have you ever discussed SRH issues with your adolescents?

Respondent: I have but not as often as I would like.

Interviewer: What topics on sexual reproductive health do you discuss with your children? What topics do you consider most important?

Respondent: My kind of job doesn’t give me enough time to discuss with my children. I’m usually really tired when I get home and I work weekends too. I only make out time to discuss really important stuff with them.

Interviewer: Name the challenges you face in talking to your adolescent children about Sex and Reproductive Health?

Respondent: I really feel shy discussing with my daughters especially on puberty and menstruation. It is the job of their mother and their school teachers.

Interviewer: What do you think will make it easier for you as a parent to discuss SRH issues with your adolescents?

Respondent: I would prefer discussing sex education with my boys rather than my daughters.

Interviewer: What SRH information would you like your adolescent to receive at school?

Respondent: Everything. As much as they possible

Interviewer: How do you think parents can be encouraged to participate in programmes on SRH in the school?

Respondent: The school should invite parents maybe, discuss and remind us of the necessity of having these discussions with our children. I think that would be really helpful.

Interviewer: Do you wish to make any comments or ask questions regarding the discussed topic?

Respondent: None.
