# Supplementary material for: Exploring barriers to parent-adolescent sexual-risk communication among adolescents in Port Harcourt Nigeria: Adolescents’ and parents’ perspective
Source: PLOS Glob Public Health. 2025 Jan 21;5(1):e0003148. doi: 10.1371/journal.pgph.0003148 (PMC11750103; doi:10.1371/journal.pgph.0003148)
Supplement: S1 File — IDI and FGD questions for adolescents and parents of adolescents respectively. (DOCX) [file pgph.0003148.s003.docx]

**S1 File. Interview Guides**

**Text A.** **In-depth Interview Guide for Parents of Adolescents**

1. What is your knowledge on Sexual and Reproductive health (SRH)?
2. Do you think that parents should discuss SRH issues with their adolescent children? Give reasons.
3. Do you think the school should discuss SRH issues with adolescent students?
4. Have you ever discussed SRH issues with your adolescents? Why or why not? [parents should be asked if they only discuss with one member of the sex or both sexes and at what age they started said discussions and also how often they do]
5. What topics are covered by parents who discuss SRH issues with their adolescents? What topics are priority?
6. Name the challenges you face in talking to your adolescent children about Sex and Reproductive Health? [Find out what challenges are peculiar to each sex]
7. What do you think will make it easier for you as a parent to discuss SRH issues with your adolescents?
8. What SRH information would you like your adolescent to receive at school?
9. How do you think parents can be encouraged to participate in programmes on SRH in the school?
10. Do you wish to make any comments or ask questions regarding the discussed topic?

**Text B. FGD Guide for Adolescents**

1. Have you ever received sex education from your parents

Probe: If Yes, Who and Where did you first receive sex education?

1. What do you think hinders your parents from effectively discussing with you on sex education?
2. Have you ever heard of Risky Sexual Behaviours? Do you know what they are with examples?
3. Do you think risky sexual behaviors can be prevented if adolescents received sex education from schools or parents?
