# Supplementary material for: Exploring barriers to parent-adolescent sexual-risk communication among adolescents in Port Harcourt Nigeria: Adolescents’ and parents’ perspective
Source: PLOS Glob Public Health. 2025 Jan 21;5(1):e0003148. doi: 10.1371/journal.pgph.0003148 (PMC11750103; doi:10.1371/journal.pgph.0003148)
Supplement: S1 Appendix — Questionnaire data collection tool for in-school adolescents aged 15–19 years. (DOCX) [file pgph.0003148.s004.docx]

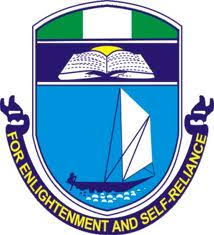


**S1 Appendix. Questionnaire for In-School Adolescents (15-19 years)**

My name is Chidinma Joycelyn Okpalaku; a postgraduate student of University of Port Harcourt School of Public Health. I am conducting a study on Exploring issues on Parent-adolescent sexual risk communication in Port Harcourt LGA in Rivers State, Nigeria. The information needed here is purely for academic purposes and your view in this study is of utmost importance to us. Do not write your name: Tick the only right response or multiple responses where applicable. The answers you give will be kept private. You are hereby invited to participate in this study and encouraged to give HONEST and ACCURATE information. Thank You

I agree to be a part of this study (tick) [ ]

Serial No Signature

**SECTION A: SOCIODEMOGRAPHIC/FAMILY CHARACTERISTICS**

| **S/N QUESTIONS RESPONSE** | | | | | | | | | | | **TICK** | | |
| --- | --- | --- | --- | --- | --- | --- | --- | --- | --- | --- | --- | --- | --- |
|  | | What is your age at your last birthday? |  | | | | | | | |  | | |
|  | | What is your Sex? | Male | | | | | | | |  | | |
|  |  |  | Female | | | | | | | |  | | |
|  | | In what class are you? | SS1 | | | | | | | |  | | |
|  |  |  | SS2 | | | | | | | |  | | |
|  |  |  | SS3 | | | | | | | |  | | |
|  | | What is your religion? | Christianity | | | | | | | |  | | |
|  |  |  | Islam | | | | | | | |  | | |
|  |  |  | Traditionalist | | | | | | | |  | | |
|  |  |  | Other (specify)________________. | | | | | | | |  | | |
|  | | What is your family type? | Monogamy | | | | | | | |  | | |
|  |  |  | Polygamy | | | | | | | |  | | |
|  |  |  | Single parent family | | | | | | | |  | | |
|  |  |  | Stepparent family | | | | | | | |  | | |
|  | | Who do you currently live with? | Two Biological parents | | | | | | | |  | | |
|  |  |  | Biological father only | | | | | | | |  | | |
|  |  |  | Biological Mother only | | | | | | | |  | | |
|  |  |  | Biological father and step mother | | | | | | | |  | | |
|  |  |  | Biological mother and step father | | | | | | | |  | | |
|  |  |  | With guardian | | | | | | | |  | | |
|  |  |  | Other family member (grandparents) | | | | | | | |  | | |
|  | | How big is your family? | Less than three (1-3) | | | | | | | |  | | |
|  |  |  | Four to Six (4-6) | | | | | | | |  | | |
|  |  |  | Seven to nine (7-9) | | | | | | | |  | | |
|  |  |  | 10 and above | | | | | | | |  | | |
|  | | What is the educational status of your father/male guardian? | No formal education | | | | | | | |  | | |
|  |  |  | Vocational training | | | | | | | |  | | |
|  |  |  | Primary | | | | | | | |  | | |
|  |  |  | Secondary | | | | | | | |  | | |
|  |  |  | Tertiary | | | | | | | |  | | |
|  | | What is the educational status of your mother/female guardian? | No formal education | | | | | | | |  | | |
|  |  |  | Vocational training | | | | | | | |  | | |
|  |  |  | Primary | | | | | | | |  | | |
|  |  |  | Secondary | | | | | | | |  | | |
|  |  |  | Tertiary | | | | | | | |  | | |
|  | | What is your father’s occupation? | Employed by the government | | | | | | | |  | | |
|  |  |  | Private employment | | | | | | | |  | | |
|  |  |  | Self-employment | | | | | | | |  | | |
|  |  |  | Unemployed | | | | | | | |  | | |
|  | | What is your mother’s occupation? | Employed by the government | | | | | | | |  | | |
|  |  |  | Private employment | | | | | | | |  | | |
|  |  |  | Self-employment | | | | | | | |  | | |
|  |  |  | Unemployed | | | | | | | |  | | |
| **SECTION B: SOURCE OF INFORMATION AND KNOWLEDGE OF REPRODUCTIVE HEALTH** | | | | | | | | | | | | | |
|  | | From whom did you learn about **puberty**?  (Puberty is a period a child’s body begins to develop and change to become adult e.g. Girls develop breast and boys develop deeper voice) | School teacher | | | | | | | | | |  |
|  |  |  | Father | | | | | | | | | |  |
|  |  |  | Mother | | | | | | | | | |  |
|  |  |  | Siblings | | | | | | | | | |  |
|  |  |  | Friends | | | | | | | | | |  |
|  |  |  | Internet/social media | | | | | | | | | |  |
|  |  |  | Other(specify)___________________ | | | | | | | | | |  |
|  | | From whom or where would you prefer to have received more information on puberty? | School teacher | | | | | | | | | |  |
|  |  |  | Father | | | | | | | | | |  |
|  |  |  | Mother | | | | | | | | | |  |
|  |  |  | Siblings | | | | | | | | | |  |
|  |  |  | Friends | | | | | | | | | |  |
|  |  |  | Internet/social media | | | | | | | | | |  |
|  |  |  | Other(specify)___________________ | | | | | | | | | |  |
|  | | Where do you mostly receive information on sexual and reproductive health? | School teacher | | | | | | | | | |  |
|  |  |  | Father | | | | | | | | | |  |
|  |  |  | Mother | | | | | | | | | |  |
|  |  |  | Siblings | | | | | | | | | |  |
|  |  |  | Friends | | | | | | | | | |  |
|  |  |  | Internet/social media | | | | | | | | | |  |
|  |  |  | Other(specify)___________________ | | | | | | | | | |  |
|  | | Have you ever received sex education? | Yes | | | | | | | | | |  |
|  |  |  | No | | | | | | | | | |  |
|  | | From whom did you receive sex education? | School teacher | | | | | | | | | |  |
|  |  |  | Father | | | | | | | | | |  |
|  |  |  | Mother | | | | | | | | | |  |
|  |  |  | Siblings | | | | | | | | | |  |
|  |  |  | Friends | | | | | | | | | |  |
|  |  |  | Social media | | | | | | | | | |  |
|  |  |  | Films/video | | | | | | | | | |  |
|  |  |  | Internet/social media | | | | | | | | | |  |
|  | | Please tick what topics/forms of sex education you received?  (**Multiple response**; tick as many as you have received) | Puberty | | | | | | | | | |  |
|  |  |  | Menstrual hygiene | | | | | | | | | |  |
|  |  |  | Abstinence | | | | | | | | | |  |
|  |  |  | Relationship | | | | | | | | | |  |
|  |  |  | Sexual abuse | | | | | | | | | |  |
|  |  |  | Contraceptive use/birth control | | | | | | | | | |  |
|  |  |  | Condom use | | | | | | | | | |  |
|  |  |  | STIs | | | | | | | | | |  |
|  |  |  | Pregnancy(prevention & consequences) | | | | | | | | | |  |
|  |  |  | Life skills | | | | | | | | | |  |
|  |  |  | Other (specify)………………………….. | | | | | | | | | |  |
| **SECTION C: RISKY SEXUAL BEHAVIOUR** | | | | | | | | | | | | | |
|  | | Have you ever had sexual intercourse? | | Yes | | | | | | | |  | |
|  |  |  |  | No | | | | | | | |  | |
|  | | How old were you when you had sexual intercourse for the first time? | | I have never had sexual intercourse | | | | | | | |  | |
|  |  |  |  | 10 years old or younger | | | | | | | |  | |
|  |  |  |  | 11 years old | | | | | | | |  | |
|  |  |  |  | 12 years old | | | | | | | |  | |
|  |  |  |  | 13 years old | | | | | | | |  | |
|  |  |  |  | 14 years old | | | | | | | |  | |
|  |  |  |  | 15 years old | | | | | | | |  | |
|  |  |  |  | 16 years old | | | | | | | |  | |
|  |  |  |  | 17 years old or older | | | | | | | |  | |
| 1. Pee | | What were the circumstances behind your **first** sexual intercourse? | | Self-decision | | | | | | | |  | |
|  |  |  |  | Curiosity | | | | | | | |  | |
|  |  |  |  | Rape | | | | | | | |  | |
|  |  |  |  | Peer pressure | | | | | | | |  | |
|  |  |  |  | Influence of alcohol/drug | | | | | | | |  | |
|  |  |  |  | Lack of parental attention | | | | | | | |  | |
|  |  |  |  | In exchange for gift/money | | | | | | | |  | |
|  |  |  |  | Other (specify)……………………… | | | | | | | |  | |
|  | | During your **life**, with how many people have you had sexual intercourse? | | I have never had sexual intercourse | | | | | | | |  | |
|  |  |  |  | 1 person | | | | | | | |  | |
|  |  |  |  | 2 people | | | | | | | |  | |
|  |  |  |  | 3 people | | | | | | | |  | |
|  |  |  |  | 4 people | | | | | | | |  | |
|  |  |  |  | 5 and above | | | | | | | |  | |
|  | | Have you ever had sex under the influence of alcohol/drug? | | Yes | | | | | | | |  | |
|  |  |  |  | No | | | | | | | |  | |
|  | | Have you ever had sex in exchange of money, gift or material again? | | Yes | | | | | | | |  | |
|  |  |  |  | No | | | | | | | |  | |
|  | | During the past **3 months**, with how many people did you have sexual intercourse? | | I have never had sexual intercourse | | | | | | | |  | |
|  |  |  |  | 1 person | | | | | | | |  | |
|  |  |  |  | 2 people | | | | | | | |  | |
|  |  |  |  | 3 people and above | | | | | | | |  | |
|  | | Have you had sex in the last **7 days**? | | Yes | | | | | | | |  | |
|  |  |  |  | No | | | | | | | |  | |
|  | | The **first time** you had sexual intercourse; did you drink alcohol or use drugs? | | I have never had sexual intercourse | | | | | | | |  | |
|  |  |  |  | Yes | | | | | | | |  | |
|  |  |  |  | No | | | | | | | |  | |
|  | | The **first** time you had sexual intercourse; did you or your partner use a condom? | | I have never had sexual intercourse | | | | | | | |  | |
|  |  |  |  | Yes | | | | | | | |  | |
|  |  |  |  | No | | | | | | | |  | |
|  | | The **last time** you had sexual intercourse with an opposite sex partner, what **one** method did you or your partner use to **prevent pregnancy**? (select only **one** response) | | I have never had sexual intercourse | | | | | | | |  | |
|  |  |  |  | No method was used | | | | | | | |  | |
|  |  |  |  | Condoms | | | | | | | |  | |
|  |  |  |  | Withdrawal | | | | | | | |  | |
|  |  |  |  | Not sure | | | | | | | |  | |
|  |  |  |  | Others (specify)……………… | | | | | | | |  | |
|  | | When you engaged in unprotected sex, what was the outcome? | | Unwanted pregnancy | | | | | | | |  | |
|  |  |  |  | Unsafe abortion | | | | | | | |  | |
|  |  |  |  | Contacted STI | | | | | | | |  | |
|  |  |  |  | Infected with HIV | | | | | | | |  | |
|  |  |  |  | No outcome | | | | | | | |  | |
|  | | Have you ever been pregnant/impregnated someone | | Yes | | | | | | | |  | |
|  |  |  |  | No | | | | | | | |  | |
|  | | During your life, with who have you had sexual contact? | | I have never had sexual contact | | | | | | | |  | |
|  |  |  |  | Male to female | | | | | | | |  | |
|  |  |  |  | Female to male | | | | | | | |  | |
|  |  |  |  | Male to male | | | | | | | |  | |
|  |  |  |  | Female to female | | | | | | | |  | |
| **SECTION D:FORMS OF SEX EDUCATION RECEIVED FROM PARENTS/CAREGIVERS.Tick YES or NO** | | | | | | | | | | | | | |
|  | Have your **parents/caregivers** ever educated you on: | | | | | **YES** | | **NO** | | | | | |
|  | Abstinence | | | | |  | |  | | | | | |
|  | Menstrual hygiene | | | | |  | |  | | | | | |
|  | Sexual intercourse | | | | |  | |  | | | | | |
|  | Reproduction and pregnancy | | | | |  | |  | | | | | |
|  | Dating and Romance | | | | |  | |  | | | | | |
|  | Birth Control | | | | |  | |  | | | | | |
|  | How to use a condom | | | | |  | |  | | | | | |
|  | Sexually Transmitted Infections (STIs) | | | | |  | |  | | | | | |
|  | Management of sexual pressure | | | | |  | |  | | | | | |
|  | How often do your Father/Mother/Guardian discuss sex education with you | | | | | Never | | | | | | | |
|  |  |  |  |  |  | Rarely | | | | | | | |
|  |  |  |  |  |  | Sometimes | | | | | | | |
|  |  |  |  |  |  | Often | | | | | | | |
|  |  |  |  |  |  | Always | | | | | | | |
|  | Who are you most comfortable with, to discuss sexual related issues? | | | | | Father | | | | | | | |
|  |  |  |  |  |  | Mother | | | | | | | |
|  |  |  |  |  |  | Siblings | | | | | | | |
|  |  |  |  |  |  | Teachers | | | | | | | |
|  |  |  |  |  |  | Peers of the same sex | | | | | | | |
|  |  |  |  |  |  | Friends | | | | | | | |
|  |  |  |  |  |  | Other(specify)___________ | | | | | | | |
|  | Where did you **first** receive information on sex education? | | | | Home | | | | |  | | | |
|  |  |  |  |  | School | | | | |  | | | |
|  |  |  |  |  | Internet/social media | | | | |  | | | |
|  |  |  |  |  | Church | | | | |  | | | |
|  | Have your Parents/Guardians ever given you information on sex education? | | | | Yes | | | | |  | | | |
|  |  |  |  |  | No | | | | |  | | | |
| **SECTION E: BARRIERS TO PARENT-ADOLESCENT SEXUAL-RISK COMMUNICATION** | | | | | | | | | | | | | |
|  | **What are some of the barriers that limit you from having an honest discussion with your parents on sex?** | | | | | | **RESPONSE** | | | | | | |
|  |  |  |  |  |  |  | **YES** | | **NO** | | | | |
|  | Fear/discomfort with discussion | | | | | |  | |  | | | | |
|  | Belief that it will initiate sex | | | | | |  | |  | | | | |
|  | Parents are ignorant or unaware | | | | | |  | |  | | | | |
|  | Parents are too busy | | | | | |  | |  | | | | |
|  | Religious factor | | | | | |  | |  | | | | |
|  | It is against Culture | | | | | |  | |  | | | | |
|  | They are too judgmental | | | | | |  | |  | | | | |
|  | I don’t trust them with my secrets | | | | | |  | |  | | | | |
|  | Poor and inaccurate knowledge about regarding sex education | | | | | |  | |  | | | | |

**This is the end of the survey. Thank you so much for your time**
