# Supplementary material for: Exploring barriers to parent-adolescent sexual-risk communication among adolescents in Port Harcourt Nigeria: Adolescents’ and parents’ perspective
Source: PLOS Glob Public Health. 2025 Jan 21;5(1):e0003148. doi: 10.1371/journal.pgph.0003148 (PMC11750103; doi:10.1371/journal.pgph.0003148)
Supplement: S1 Checklist — Code book for FGD of in-school adolescents and IDI of parents of adolescents respectively. (DOCX) [file pgph.0003148.s005.docx]

**S1 Checklist. Code Books**

**File A. Focus Group Discussion Code Book for Adolescents**

Sex education_Yes

Sex education_No

Sex education_frequency

Sex education_source

Barrier_Fear

Barrier_Discomfort

Barrier_Sex education_leads to sex

Barrier_Ignorance

Barrier_Parents not availiable

Barrier_parents too busy

Barrier_religious

Barrier_cultural

Barrier_parents too judegmental

Barrier_parents lack knowledge

Barrier_lack trust

Prevention_RSB

**File B. In-depth Interview Code Book for Parents**

Sex education_knowldege

Perception_sex education_positive

Perception_sex education_negative

Barrier_shame

Barrier_cultural

Barrier_religious

Barrier_increseases sexual experiementations

Barrier_children too young

Barrier_not enough time

Barriesr_lack of education

Faciliator_adolescent initiate education

Faciliataor_adolescent maturity

Facilitator_education

Facilitator_parents involvement_school

Facilitator_religion

Facilitator_cultural
