# Supplementary material for: Exploring barriers to parent-adolescent sexual-risk communication among adolescents in Port Harcourt Nigeria: Adolescents’ and parents’ perspective
Source: PLOS Glob Public Health. 2025 Jan 21;5(1):e0003148. doi: 10.1371/journal.pgph.0003148 (PMC11750103; doi:10.1371/journal.pgph.0003148)
Supplement: Attachment — Submitted filename: Review_Adolescent and parent perspective.docx [file pgph.0003148.s007.docx]

Good day,

I am grateful for the opportunity to be one of the reviewers of this manuscript and I also wish to commend the authors seriously for the efforts put in to develop this article. The topic: Exploring Barriers to Parent-Adolescent Sexual-Risk Communication among Adolescents in Port Harcourt Nigeria: Adolescents’ and Parents’ Perspective is an interesting one, and insights have been revealed.

However, the authors still have some touch-ups to do. A clear justification was not given regarding the need for the study, and what makes it different from other studies on this topic. Also, a lot of copy editing need to be done to correct grammatical and spelling errors. Capital letters and abbreviations should be maintained where appropriate.

In addition, there are a lot of repetitions especially in the result section which can bore its reader’s. In the discussion section, suggestions regarding using more recent studies and studies conducted in West Africa and Nigeria have been made in other to contextualize the study. Lastly, the introductory paragraph of the conclusion looks like the authors are repeating the results, hence be rewritten.

Title: Good

Abstract: Good

Background: The background can be worked on to reduce repetition of the implications of risky sexual behaviour. Most importantly justify the need for the study. How does this study differ from other studies on this topic? What gap in literature do you aim to fill?

Line 40 -42: Sexual curiosity and sexual desire are two different words. Hence, the previous statement those not transcend into the next.

Line 45: Adolescent that are sexually active means that the adolescents are engaging in sexual activity already. Early sexual activity should be struck out from the list of the implications.

Line 48 & 49: The issues should be listed out; using the term several issues is vague.

Line 53: Other non-risky sexual behaviours e.g. engaging in safe sex and abstinence still marriage should be included in the list.

Line 59-60: “Problems associated with” is more appropriate.

Line 78-79: from both the adolescent and parents perspective

Methods: Was permission received from the Ministry of Education/health? This was not stated.

Line 148: Recruitment of participants

Results: Correct grammatical errors in quotes. Edit all “I” to be in capital letter. Once a phrase has been abbreviated, stick to the abbreviations in the later text e.g. SRH. This was also repeated in the discussion section.

Line 257: some from my friends too.

Line 267: that is why I am asking…

Line 271: strike out “health”

Lines 278, 279 & 280 should come at the beginning of the section for parents are too judgemental. Same should be replicated for subsequent sections.

Line 305: the first time

Line 518: may not understand the importance of the discussion

Line 528 & 529: is a repetition. The point has been earlier explained at the beginning of the section

Line 542: “conversation” not “conservation”

Line 562, 563 & 564: and repetition

Discussion: To contextualize the study, more West African and Nigerian studies should be used for Comparism. Also, take note of the dates to use most recent studies.

Line 663: “increase” not “increases”

Conclusion: The introductory part of the conclusion looks more like the result section. Hence, should be rewritten.

References: Well arranged
