# Supplementary material for: Exploring barriers to parent-adolescent sexual-risk communication among adolescents in Port Harcourt Nigeria: Adolescents’ and parents’ perspective
Source: PLOS Glob Public Health. 2025 Jan 21;5(1):e0003148. doi: 10.1371/journal.pgph.0003148 (PMC11750103; doi:10.1371/journal.pgph.0003148)
Supplement: Attachment — Submitted filename: Response to Reviewers.docx [file pgph.0003148.s008.docx]

Chidinma Joycelyn Okpalaku
University of Port Harcourt

Choba, Rivers State
chidinma.okpalaku@gamil.com
1/10/2024

The Editor-in-chief
PLOS Global Public Health

Dear Dr. Kyobutungi,

**RESPONSE TO REVIEWERS**

Thank you for the opportunity to revise and resubmit our manuscript titled “Exploring Barriers to Parent- Adolescent Sexual Risk Communication among Adolescents in Port Harcourt, Nigeria: Adolescents’ and Parents’ perspective” (PGPH-D-24-00489). We appreciate the thoughtful comments and constructive feedback provided by you and the reviewers. We have carefully considered each point raised and have made the following revisions to address the concerns.

We have attached a point-by-point response to the reviewers’ comments: We believe that these revisions have significantly improved the manuscript and addressed the reviewers’ concerns. We have attached a marked-up copy of the manuscript highlighting the changes made, as well as an unmarked copy for your review.

Thank you again for your valuable feedback and for considering our revised manuscript. We look forward to your response.

Sincerely,
Chidinma J. Okpalaku

| **REVIEWER** | **COMMENT** | **PREVIOUS LINE NUMBER** | **CURRENT LINE NUMBER** | **RESPONSE** |
| --- | --- | --- | --- | --- |
| Reviewer 2 | Do you mean quantitative? | 16 | 16 | I have attended to it |
| Reviewer 2 | 21% does not quite represent a quarter | 21 | 21 | I have attended to it |
| Reviewer 3 | Sexual desire and sexual curiosity | 40-42 | 41-42 | I have attended to it |
| Reviewer 3 | Early sexual activity should be struck out | 45-46 | 47-48 |  |
| Reviewer 3 | The issues should be listed out; using the term several issues is vague. | 48-49 | Nil | Line was struck out to avoid repetition |
| Reviewer 3 | Other non-risky sexual behaviours e.g. engaging in safe sex and abstinence still marriage should be included in the list. | 51 | 53-54 | I have attended to it |
| Reviewer 3 | “Problems associated with” is more appropriate | 57 | 60 | I have attended to it |
| Reviewer 3 | from both the adolescent and parents perspective | 78-79 | 77-78 | I have attended to it |
| Reviewer 2 | You left out adolescent perspective which is in your title | 85 | 83 | I have attended to it |
| Reviewer 3 | Was permission received from the Ministry of Education/health? This was not stated | 132 | 134 | I have attended to it |
| Reviewer 2 | What about the JSS3 you mentioned earlier? | 138-139 | 138-139 | Added |
| Reviewer 3 | Recruitment of participants, Is this a pilot study? | 148 | 148 | Reversed |
| Reviewer 2 | ….self-administered and not interviewer….. | 151-152 | 151-152 | Edited |
| Reviewer 2 | You didn’t describe the settings in which FGD was done | 153 | 152-154 | Added |
| Reviewer 2 | This is for section on ethical consideration | 154-158 | 177-183 | I have attended to it |
| Reviewer 2 | SPSS is not for qualitative. Did you mean quantitative? | 172 | 167 | I have attended to it |
| Reviewer 2 | Provide the ethical approval number | 180 | 175 | I have attended to it |
| Reviewer 2 | …of the respondents | 209, 215, 230 | 208, 215, 230 | Done |
| Reviewer 2 | It would have been nice to see whether any of the socio-demographic factors was associated with receiving sexual ….. | 235 |  | It’s not in line with the study objectives |
| Reviewer 2 | “ “ for all quotes | 250, 252, 255, 257…………….. | 250, 252, 255, 257……………. | I have attended to it |
| Reviewer 2 | our | 251 | 251 | I have attended to it |
| Reviewer 2 | gain | 280 | 279 | I have attended to it |
| Reviewer 3 | i to I in quotes | 273, 275, 418,525, 559 | 276,278, 422, 535, 568 | I have attended to it |
| Reviewer 2 | Recast | 345 | 347 | I have attended to it |
| Reviewer 2 | The discussion section should follow an inverted funnel shape. You review studies from local-national-regional | 575-588 | 581-601 | I have attended to it |
| Editor | Authors should consider reworking the introduction—there is a lot of repetition that could be reduced. | 50 – 79 | 48-74 | Introduction reworked, repetitions reduced |
| Editor | It would also be helpful to situate parent-youth communication and youth SRH in the Nigerian context. What is the situation in Nigeria, and what is the gap in the literature that this  study will fill? |  | 63-67 | Done |
| Editor | - I would also encourage the authors to avoid overgeneralizing in their review of the literature in the introduction (for example, “Parental and societal distaste to sex education hasn’t changed over time…”—where is this the case? In certain countries/settings, there is broad support for school-based sex education.) | 73-74 |  | Added |
| Editor | In the methods section, it would help to explain why the explanatory sequential design was chosen, as opposed to other approached | 105-106 | 97-98 | Done |
| Editor | In addition, I would encourage the authors to review the COREQ guidelines for reporting qualitative studies, and use this to help in structuring the qualitative component of the manuscript. More information is needed on the qualitative component of the study (how participants were identified and recruited, why IDIs vs. FGDs, qualitative sample size, how thematic analysis was conducted and by whom, etc.), and aligning your revisions with COREQ or a similar qualitative reporting guideline will help. |  | 97-98  110-117  150-152  134-135  165-167  181-185 | Done |
| Editor | Please include copies of the interview and discussion guides, qualitative codebook(s), and survey instruments as supplemental files. |  |  | Attached as supplementary files |
| Editor | Under the ethical consideration section, it would be helpful to clarify why school principals served as guardians for providing consent, instead of parents. |  | 190-197 | Done |
| Editor | - The results are extremely long. I would encourage the authors to consider re-organizing and streamlining the results. In addition, it would be helpful to provide the readers with an introductory paragraph to the results to orient them. This would also be a good place to note the primary qualitative themes identified. |  | 245 - 467 | Done |
| Editor | - The qualitative results could use a little more “scaffolding” or explanation. Right now, it reads a little bit like a list of themes and quotes, requiring the reader to make connections. |  | 245 - 467 | Done |
| Editor | - The integration of quant and qual data should be clarified in the methods and results. Right now, it isn’t clear whether/how these were integrated in line with the named mixed methods design. |  | 99-100  473-474 | Clarified |
| Editor | - The discussion is also very long and could use some reorganizing as suggested by the reviewers. |  | 470-577 | Discussion summarized |
| Editor | - In the limitations section, you note that some of the youth were shy discussing the topic, but were assured of confidentiality. However, confidentiality cannot be assured in a FGD, which is why FGDs are often not used for discussing sensitive topics and experiences. I would suggest that the authors include this as a limitation. The limitations of the quantitative data also need to be addressed. |  | 588-592 | Added |
