# Supplementary material for: Exploring barriers to parent-adolescent sexual-risk communication among adolescents in Port Harcourt Nigeria: Adolescents’ and parents’ perspective
Source: PLOS Glob Public Health. 2025 Jan 21;5(1):e0003148. doi: 10.1371/journal.pgph.0003148 (PMC11750103; doi:10.1371/journal.pgph.0003148)
Supplement: Attachment — Submitted filename: Response_to_Reviewers_auresp_2.docx [file pgph.0003148.s009.docx]

Chidinma Joycelyn Okpalaku
University of Port Harcourt

Choba, Rivers State
chidinma.okpalaku@gmail.com
05/12/2024

The Editor-in-chief
PLOS Global Public Health

Dear Dr. Kyobutungi,

**RESPONSE TO REVIEWERS**

Thank you for the opportunity to revise and resubmit our manuscript titled “Exploring Barriers to Parent- Adolescent Sexual Risk Communication among Adolescents in Port Harcourt, Nigeria: Adolescents’ and Parents’ perspective” (PGPH-D-24-00489). We appreciate the thoughtful comments and constructive feedback provided by you and the reviewers. We have carefully considered each point raised and have made the following revisions to address the concerns.

We have attached a point-by-point response to the reviewers’ comments: We believe that these revisions have significantly improved the manuscript and addressed the reviewers’ concerns. We have attached a marked-up copy of the manuscript highlighting the changes made, as well as an unmarked copy for your review.

Thank you again for your valuable feedback and for considering our revised manuscript. We look forward to your response.

Sincerely,
Chidinma J. Okpalaku

| **Reviewer** | **Comment** | **Previous Line Number** | **Current Line Number** | **Response** |
| --- | --- | --- | --- | --- |
| Editor | The discrepancy between the sample size calculated vs. the actual sample size needs to be addressed as noted by the reviewer's comments below. They offer several suggestions for addressing this, and if not possible, then this should be addressed in the limitations section of the manuscript. | 205-206 | 206-207, 574-578 | I have addressed this as a limitation of the study. |
| Reviewer 2 | Your study respondents (329) are below your estimated minimum sample size (394) and should be addressed. The implications are far-reaching. I advise you to apply a correction formula to your estimated sample size, which should reduce your sample size. So that the 329 can be valid. | 205-206 | 206-207, 574-578 | This has been addressed as a limitation of the study |
|  |  |  |  |  |

Journal Requirements: References 31 was removed as it was same as reference 20. References 36 and 37 were both removed as there were never referenced in the manuscript itself.
